# Maternal, Behavioral, and Environmental Factors Associated with *Toxoplasma gondii* Infection in Pregnancy in Italy: A Case–Control Study

**DOI:** 10.3390/diagnostics16040606

**Published:** 2026-02-19

**Authors:** Adele Vasta, Francesca Arcieri, Stella Borza, Gregorio Volpe, Martina Rivabene, Valentina D’Ambrosio, Maria Caterina Corigliano, Daniele Di Mascio, Giuseppe Rizzo, Antonella Giancotti

**Affiliations:** 1 Department of Maternal and Child Health and Urological Sciences, Sapienza University of Rome, Piazzale Aldo Moro, 5, 00185 Rome, Italy; adele.vasta@uniroma1.it (A.V.); francesca.arcieri@uniroma1.it (F.A.); stella.borza@uniroma1.it (S.B.); gregorio.volpe@uniroma1.it (G.V.); daniele.dimascio@uniroma1.it (D.D.M.); giuseppe.rizzo@uniroma1.it (G.R.); 2 Faculty of Pharmacy and Medicine, Sapienza University of Rome, Piazzale Aldo Moro, 5, 00185 Rome, Italy; martinarivabene99@gmail.com; 3 Azienda Ospedaliero-Universitaria Policlinico Umberto I, Viale del Policlinico, 155, 00161 Roma, Italy; dr.valentina.dambrosio@gmail.com (V.D.); m.corigliano@policlinicoumberto1.it (M.C.C.)

**Keywords:** maternal toxoplasmosis, maternal risk factors, pregnancy

## Abstract

**Background:** Congenital toxoplasmosis remains a significant cause of fetal morbidity worldwide. This case–control study aimed to identify sociodemographic, dietary, and behavioral factors associated with *Toxoplasma gondii* infection during pregnancy in Italy by comparing infected women with seronegative controls, and to evaluate modifiable risk behaviors and treatment-related outcomes among affected patients. **Methods:** This case–control study included 201 pregnant women (100 with *T. gondii* infection and 101 seronegative controls). Participants completed a structured questionnaire on sociodemographic factors, diet, environmental exposures, and preventive behaviors. Clinical data were collected for infected women and analyzed using SPSS v27.0. **Results:** Sociodemographic and obstetric characteristics did not differ between groups. Infected women were more likely to live in rural areas (*p* < 0.001), have a lower educational level (*p* = 0.009), consume unpasteurized dairy products and cured meats (*p* < 0.05), purchase food from farmers or butchers (*p* = 0.01), and do not check food preparation practices when eating outside the home. High-risk animal-related behaviors were also more common among cases (*p* < 0.001). Despite similar baseline awareness, adherence to preventive measures was lower among cases; most infections were diagnosed in the first trimester and treated with spiramycin with good tolerability. **Conclusions:** Maternal toxoplasmosis is mainly associated with modifiable behavioral and environmental factors, underscoring the need for targeted, preconception counseling to reduce the risk of congenital infection.

## 1. Introduction

Congenital toxoplasmosis, one of the most significant congenital infections worldwide, represents a major cause of miscarriage, fetal death, and a wide spectrum of neonatal clinical manifestations of varying severity, potentially leading to lifelong neurological and ophthalmological complications. The global incidence of congenital toxoplasmosis is estimated at 0.15 cases per 10,000 live births, while data from the EUROTOXO program indicate wide variability across Europe, ranging from 0.7 cases per 10,000 live births in Sweden to 13 cases per 10,000 in Germany, with substantial geographic variability influenced by climatic and socioeconomic factors [1,2].

Maternal infection may be acquired through multiple transmission routes. The most common is dietary exposure, particularly through the consumption of undercooked meat, which represents a major source of infection in many countries and is estimated to account for 30–60% of cases during pregnancy [3]. Other relevant transmission routes include the ingestion of sporulated oocysts from contaminated soil, cat litter, unwashed or unpeeled fruits and vegetables, exposure during gardening activities, and consumption of contaminated water [3]. In addition, certain insects, such as the housefly (*Musca domestica*) and the oriental latrine fly (*Chrysomya megacephala*), may act as mechanical vectors by transferring viable oocysts from cat feces to food for up to two days [4]. Less common but documented routes of transmission include blood transfusion and organ transplantation [3].

The risk of vertical transmission increases with advancing gestational age, whereas the severity of fetal disease is inversely related to the timing of maternal infection [5,6]. A large French cohort study reported maternal–fetal transmission rates of 2.2% at 6 weeks, 23.0% at 18 weeks, and 56.0% at 30 weeks of gestation [7]. Conversely, an Italian study demonstrated a very low transmission risk (0.13%) in women treated with spiramycin throughout pregnancy who had positive IgM and IgG with low-to-intermediate IgG avidity in the first trimester but no documented seroconversion [8].

During pregnancy, the diagnosis of *T. gondii* infection is primarily based on serological testing, including the detection of specific IgG and IgM antibodies. While IgG positivity generally indicates past infection, IgM positivity may suggest recent infection but requires cautious interpretation because IgM antibodies can persist for prolonged periods. IgG avidity testing is therefore used to help distinguish recent primary infection from infection acquired before pregnancy and to guide clinical management [5].

Clinical outcomes range from spontaneous abortion and intrauterine fetal death to permanent neurological and visual sequelae or completely asymptomatic forms [5,6]. Classically, congenital disease is characterized by the Sabin triad of chorioretinitis, hydrocephalus or microcephaly, and intracranial calcifications, later expanded to include seizures. Approximately 20% of affected neonates develop clinically evident manifestations at birth or during early infancy [9]. These data are of critical importance in obstetric counseling, as they allow for accurate risk stratification based on the timing of infection and the therapeutic interventions implemented.

Consequently, primary prevention, based on the adoption of appropriate behaviors during pregnancy, represents the main strategy for reducing the risk of infection [10]. However, the actual level of knowledge regarding preventive measures among pregnant women, particularly those who acquire the infection during pregnancy, has not yet been fully characterized.

The primary objective of this case–control study was to identify socio-demographic, dietary, and behavioral factors associated with *T. gondii* infection during pregnancy by comparing women diagnosed with toxoplasmosis with healthy seronegative controls. The secondary objectives were to further characterize modifiable risk behaviors, as reflected by differences in preventive practices and lifestyle habits between cases and controls, and to assess treatment-related adverse effects and adherence to therapy among the pregnant women diagnosed with toxoplasmosis.

## 2. Materials and Methods

### 2.1. Study Design and Setting

This observational case–control study was conducted at the Obstetric Day Hospital of the Department of Maternal and Child Health and Urological Sciences, Policlinico Umberto I, Sapienza University of Rome, Italy. Data were collected between January 2023 and August 2025 (for cases) and from May 2025 to August 2025 (for controls). During this period, all pregnant women who voluntarily agreed to participate were invited to be enrolled in the study.

Participation was proposed at the first consultation, and enrollment occurred after confirmation of serological status, allowing for the classification of women as cases or controls. Written informed consent was obtained from all participants prior to enrollment.

An anonymous, structured questionnaire consisting of predefined multiple-choice items was administered. Anonymity was ensured to promote accurate and reliable responses. The use of predefined answers allowed a more objective assessment of the participants’ knowledge and beliefs regarding toxoplasmosis. The questionnaire was developed based on previously published studies and required approximately 20 min to complete.

### 2.2. Study Population

The study population consisted of pregnant women attending the Obstetric Day Hospital during the study period. A total of 201 pregnant women were included in the study, in particular 100 women with serologically confirmed *T. gondii* infection (cases) and 101 seronegative pregnant women with physiological pregnancies (controls).

- The case group included women with a serologically confirmed diagnosis of *T. gondii* infection during pregnancy or the periconceptional period, recruited from January 2023 to August 2025.
- The control group consisted of pregnant women with physiological pregnancies and negative serology for *T. gondii*, recruited from May 2025 to August 2025, in the same clinical setting to ensure comparability.

#### 2.2.1. Inclusion Criteria

- The case group included only pregnant women with confirmed *T. gondii* infection acquired during pregnancy or the periconceptional period, defined by the detection of IgM positivity during pregnancy or in the periconceptional period. In all women included in the case group, IgG antibodies and the IgG avidity index were systematically evaluated to confirm the timing of infection);
- Physiological pregnancy with negative *T. gondii* serology (control group);
- Age ≥ 18 years;
- Written informed consent.

#### 2.2.2. Exclusion Criteria

- Women presenting with negative IgM, positive IgG, and a high IgG avidity index, consistent with infection acquired before pregnancy, were excluded from the study.
- Cognitive or language limitations preventing comprehension or completion of the questionnaire;
- Age < 18 years.

### 2.3. Data Collection and Questionnaire

The questionnaire aimed to assess the participants’ knowledge of *T. gondii* infection, previous screening practices, awareness of transmission routes, and behaviors adopted to prevent infection during pregnancy. The questionnaire was administered in a separate setting by a trained operator who was not involved in routine clinical care, independently of the consulting physicians, in order to minimize information bias.

Demographic and socio-demographic data were collected, including age, ethnicity, parity, educational level, employment status, marital status, and type of antenatal care provider. Obstetric history was also recorded, including the number of previous pregnancies, spontaneous abortions, mode of conception, and setting of the first obstetric visit.

All participants, including both cases and controls, were asked standardized questions regarding dietary and hygienic behaviors. For women in the case group, information referred to the period immediately preceding the diagnosis of *T. gondii* infection, whereas for seronegative controls, it referred to a comparable reference period during pregnancy, in order to standardize exposure assessment and minimize potential recall bias. These included the consumption of raw or undercooked meat, raw vegetables, unpasteurized milk, and meals consumed outside the home, as well as food preparation and cooking practices.

Environmental exposure was assessed in all participants by investigating contact with farm animals, gardening or agricultural activities, methods of purchasing and washing vegetables, use of organic products, and cohabitation with cats. In participants reporting cat ownership, litter box management, household hygiene practices, and the animal’s feeding habits were specifically evaluated.

Additional data were collected from the case group regarding the clinical history of *T. gondii* infection, including gestational age at diagnosis, frequency of serological screening, suspected route of transmission, pharmacological treatment, adherence to therapy, and the occurrence of treatment-related adverse effects.

Women with confirmed primary *Toxoplasma gondii* infection were managed according to a dedicated clinical protocol, which included periodic serological monitoring (IgG, IgM, and IgG avidity testing), infectious disease specialist consultations, and serial ultrasound examinations every 4–6 weeks. Amniocentesis was offered and performed 6–8 weeks after documented seroconversion and not before the 20th week of gestation, with real-time PCR analysis of amniotic fluid for the detection of *T. gondii* DNA. Among all women included in the follow-up, only one case showed a positive PCR result on amniotic fluid; however, the newborn did not present clinical signs or congenital anomalies attributable to congenital *T. gondii* infection at birth or during subsequent follow-up.

### 2.4. Ethical Considerations

All data were collected and stored in fully anonymized form in accordance with Regulation (EU) 2016/679 (General Data Protection Regulation, GDPR). The study was conducted in compliance with the principles of the Declaration of Helsinki. Participation was voluntary, and confidentiality was ensured throughout all phases of the study.

### 2.5. Statistical Analysis

Data were managed and analyzed using IBM SPSS Statistics software (version 27.0; IBM Corp., Armonk, NY, USA). Categorical variables were expressed as absolute frequencies and percentages, while continuous variables were reported as mean ± standard deviation. Comparisons between women with *T. gondii* infection and controls were performed using Pearson’s chi-square (χ^2^) test and Fisher’s exact test. The strength of associations between categorical variables was assessed using Cramer’s V. Student’s *t*-test for independent samples was used to compare maternal age between groups, with the homogeneity of variances evaluated using Levene’s test. All tests were two-tailed, and a *p*-value < 0.05 was considered statistically significant. Multivariable analyses were not performed, as the study was designed primarily as an exploratory analysis aimed at identifying potential associations between individual sociodemographic, behavioral, and environmental factors and *Toxoplasma gondii* infection. Given the sample size and the number of variables considered, multivariable modeling was not undertaken in the present study.

## 3. Results

### 3.1. Characteristics of the Study Population

Baseline characteristics of the study population are summarized in Table 1. The mean maternal age was 34.1 ± 5.3 years, with no statistically significant difference between cases and controls. The majority of participants were of Caucasian ethnicity (189/201, 94.0%), while the remaining women were of Asian, African, South Asian, or South American ethnicities.

Regarding obstetric history, 137 women (68.2%) were nulliparous, while 64 (31.8%) were multiparous, with no significant difference between the two groups. No statistically significant differences were observed between cases and controls with respect to marital status and the method of conception, which was predominantly spontaneous in both groups, with a smaller proportion of pregnancies conceived through homologous or heterologous medically assisted reproduction (*p* = 0.22).

Overall, 163 participants (81.1%) resided in urban areas, whereas 38 women (18.9%) lived in rural settings; residence in rural areas was significantly more frequent among cases (*p* < 0.001). Regarding the educational level, most women had completed secondary education or held a university degree. Specifically, 104/201 women (51.7%) had a university degree, while 72/201 (35.8%) had completed secondary school. After recategorization of educational level into three groups: low education (primary and lower secondary education), medium education (upper secondary education), and high-level education (university degree, PhD, and second-level master), a statistically significant difference was observed between the cases and controls (*p* = 0.0085, Cramer’s V = 0.22). Employment status did not differ significantly between the cases and controls, with most women being employed.

In addition, a significant difference in antenatal care setting was observed between groups, with a higher proportion of women followed by private gynecologists in the case group (*p* < 0.001).

### 3.2. Screening Practices and Knowledge of Toxoplasmosis

Screening practices and knowledge related to toxoplasmosis are summarized in Table 2. No significant differences were observed between the cases and controls regarding preconception toxoplasmosis screening, which was performed in a similar proportion of women in both groups (*p* = 0.987). Likewise, baseline awareness of toxoplasmosis before pregnancy did not differ significantly between the cases and controls (*p* = 0.252), nor did awareness of preventive measures (*p* = 0.174). The main sources of information were healthcare professionals, followed by Internet or social media and family or friends, with no significant differences between groups (*p* = 0.15).

In contrast, significant differences emerged in behavioral changes following awareness of infection. Women in the control group were significantly more likely to report behavioral changes after becoming aware of toxoplasmosis compared with cases (*p* < 0.001). Conversely, the proportion of women who reported recommending preventive measures to others did not differ significantly between groups (*p* = 0.14).

### 3.3. Dietary Habits During Pregnancy

Dietary habits at the time of infection for cases and during pregnancy for the controls are summarized in Table 3. Overall dietary patterns (omnivorous vs. vegetarian) did not differ significantly between groups (Fisher’s exact test, *p* = 0.68), nor did the consumption of raw or undercooked meat (*p* = 0.97).

In contrast, women in the case group reported a significantly higher consumption of cured meats at least once per week, whereas the controls more frequently reported never consuming these products (*p* < 0.001). In addition, cases reported a significantly higher consumption of unpasteurized milk or dairy products compared with the controls (*p* < 0.005).

Consumption of fresh vegetables did not differ significantly between cases and controls, with a comparable frequency of intake across groups (*p* = 0.27). Similarly, no statistically significant differences were observed in the consumption of frozen vegetables between the cases and controls; although slight variations in intake were observed, these differences did not reach statistical significance (*p* = 0.077).

A significant difference emerged regarding food purchasing practices for items such as vegetables and meat. Women in the case group more frequently reported purchasing food directly from farmers or butchers compared with the controls, and this difference was statistically significant (*p* = 0.0023).

No significant difference was observed in the frequency of meals consumed outside the home between the cases and controls (*p* = 0.10). Consistent with this finding, the eating behaviors related to meals consumed in restaurants, bars, or takeaway, as well as food safety practices, are detailed in Table 4. When eating away from home, the controls more frequently reported always checking the cooking of meat and vegetables compared to women with *T. gondii* infection, resulting in a statistically significant difference between groups (*p* = 0.001).

### 3.4. Animal Exposure and Cat-Related Behaviors

Animal exposure and cat-related behaviors are summarized in Table 5. A significant association was observed between *T. gondii* infection and exposure to animals and rural environments. Contact with farm animals or rural settings was significantly more frequent among the cases than the controls (*p* < 0.001). Similarly, cohabitation with cats was reported more often in infected women compared with the controls (*p* = 0.0049).

With regard to litter box management, significant differences were observed between groups (*p* = 0.0014). Women in the case group more frequently reported direct or indirect exposure to cat litter, whereas the controls more often reported no exposure to litter boxes or cats.

Furthermore, feeding cats with raw or undercooked meat was reported exclusively among cases (6% vs. 0%), with this difference reaching statistical significance (Fisher’s exact test, *p* = 0.014).

Overall, these findings highlight a strong association between *T. gondii* infection and specific animal-related exposures, particularly contact with farm environments, cat ownership, litter box handling, and high-risk feeding practices.

### 3.5. Timing and Suspected Source of Infection, and Treatment-Related Adverse Effects Among Infected Women

Among women with *T. gondii* infection, the diagnosis was made predominantly during the first trimester of pregnancy (80%), with lower proportions diagnosed during the second one (11%), in the periconceptional period (5%), or at unspecified gestational ages (4%).

Moreover, information regarding the suspected source of infection was collected. The most frequently reported presumed route of transmission was the consumption of raw or undercooked meat (40%), followed by contact with animals (16%) and the consumption of raw vegetables (16%). Less frequently reported hypotheses included blood transfusions (3%), ingestion of contaminated water (1%), and other possible food-related exposures (16%).

Regarding pharmacological treatment, most women (90%) reported receiving spiramycin, while 8% were treated with pyrimethamine in addition to sulfadiazine; only 2% reported not having received any treatment. Among the treated patients, therapeutic adherence was high: 75% reported adherence for more than 90% of the prescribed treatment duration, 15% reported adherence between 75–90%, 9% between 50–75%, and only 1% reported a complete lack of adherence.

Finally, among the treated women, 31% reported the occurrence of spiramycin-related adverse effects. The most frequently reported side effects were gastrointestinal symptoms (22%), followed by cutaneous reactions (6%) and the elevation of liver enzymes (3%). Less frequently reported adverse events included central nervous system symptoms (3%), systemic symptoms such as fever or fatigue (2%), and genitourinary disorders (2%). Anemia and musculoskeletal symptoms were reported sporadically (1%).

## 4. Discussion

This case–control study provides a comprehensive evaluation of sociodemographic characteristics, knowledge, preventive behaviors, dietary habits, and environmental exposures among pregnant women with *T. gondii* infection compared with seronegative controls. By directly comparing infected and non-infected women within the same clinical setting, our findings indicate that maternal toxoplasmosis is more strongly associated with modifiable behavioral and environmental factors than with baseline demographic or obstetric characteristics alone.

In our cohort of 201 pregnant women, maternal age, parity, marital and employment status did not differ significantly between the cases and controls, suggesting that these variables did not independently influence the risk of infection. Likewise, the method of conception was comparable between groups. These results support the notion that toxoplasmosis risk during pregnancy appears to be largely independent of obstetric history and reproductive characteristics, and instead primarily related to exposure-driven factors. A significant difference was observed in educational level between groups, with a higher proportion of infected women among those with lower educational attainment (*p* = 0.009, Cramer’s V = 0.22). This finding suggests that educational level may represent a relevant socioeconomic determinant of *T. gondii* infection risk, likely through its influence on health literacy, access to preventive information, and the ability to adopt effective preventive behaviors, as consistently reported in the literature, including studies by Martini et al. [11,12].

The association with educational level does not necessarily contradict the absence of differences in employment status, as these variables capture distinct and non-overlapping dimensions of socioeconomic status. While employment status reflects occupational engagement, educational attainment may more directly shape risk perception and preventive decision-making. In this respect, our findings partially contrast with previous studies, such as that by Qadeer et al., which identified age and employment status as key determinants of infection risk, possibly reflecting contextual differences in healthcare access and health education [13].

In contrast, other sociodemographic variables were significantly associated with infection status. Firstly, residence in rural areas emerged as one of the strongest determinants of *T. gondii* infection, with infected women being significantly more likely to live in rural settings than the controls (*p* < 0.001). This observation is consistent with previous European studies identifying rural residence as a proxy for increased environmental exposure to contaminated soil, farm animals, and less regulated food production chains [14,15,16].

Despite similar baseline awareness and sources of information between groups, differences emerged in the translation of knowledge into preventive behaviors. The higher proportion of women followed by private gynecologists among cases may represent an important point for reflection. In hospital-based settings, antenatal care is often delivered according to standardized protocols and multidisciplinary pathways, which may facilitate more consistent preventive counseling and foster a more interactive communication process, encouraging patients to ask questions and allowing for the verification of message comprehension. In contrast, antenatal care in private practice is frequently based on an individualized model, in which time constraints or variability in counseling approaches may limit opportunities for extended dialogue or for systematically assessing the patients’ understanding of preventive recommendations. Although this interpretation is exploratory, it suggests that differences in care organization and communication dynamics—rather than the care setting itself—may contribute to the observed association.

In the present study, awareness was defined as self-reported knowledge of *T. gondii* infection and its possible transmission routes, reflecting a basic or superficial level of awareness rather than a comprehensive understanding of effective prevention. These findings suggest that awareness alone may not be sufficient to prevent infection. Indeed, although most pregnant women reported being aware of *T. gondii* infection, this awareness did not consistently translate into accurate risk perception or appropriate preventive behaviors, with fewer than one quarter demonstrating an adequate understanding of effective preventive measures, as also reported by Fernicola et al. and Smereka et al. [17,18].

In contrast, a significant difference emerged in behavioral changes following awareness of infection, with controls more frequently reporting modifications in preventive behaviors (*p* < 0.001). This finding highlights a gap between knowledge and effective behavioral change. Although most women reported recommending preventive measures to others, this behavior did not differ significantly between groups, indicating that information sharing does not necessarily translate into personal risk reduction.

Dietary habits represented a major differentiating factor between groups. Although overall dietary patterns did not differ significantly, with both groups mainly adhering to an omnivorous diet, infected women reported a higher consumption of specific high-risk foods, particularly cured meats and unpasteurized dairy products, or purchasing food directly from farmers or livestock breeders. Notably, when eating out, infected women were less likely to verify the adequate cooking of meat and vegetables. Taken together, these findings suggest that exposure risk is influenced not only by the type of food consumed but also by food sourcing and handling practices.

Conversely, no significant differences were observed in the consumption of fresh or frozen vegetables, indicating that vegetable intake itself may not represent a major risk factor when appropriate washing procedures are followed.

Interestingly, the lack of association between infection and raw or undercooked meat consumption contrasts with earlier European case–control studies [19,20]. This discrepancy may reflect improvements in food safety regulations, changes in meat production practices, and increased prenatal counseling on this specific risk factor. Consequently, traditionally recognized high-risk foods may now play a less prominent role, while other exposure, such as unpasteurized dairy products, may represent emerging and underappreciated sources of infection. Recent reviews support this evolving epidemiological pattern and highlight the need for updated, context-specific dietary recommendations [11,18,21].

A clear association emerged between *T. gondii* infection and animal-related exposures. Women in the case group reported significantly more frequent contact with farm animals or rural environments, higher rates of cohabitation with cats, and greater exposure to litter box handling compared with the controls. In particular, direct or indirect contact with cat litter and feeding cats with raw or undercooked meat were significantly more common among cases, supporting the role of feline-related practices as important risk factors for infection. These findings are consistent with the established transmission pathways of *T. gondii* and highlight the relevance of both environmental exposure and specific behavioral practices in determining infection risk during pregnancy [3,13].

From a clinical perspective, most infections were diagnosed during the first trimester, a period associated with lower transmission rates but potentially more severe fetal outcomes. Treatment was predominantly based on spiramycin rather than pyrimethamine and sulfadiazine, with high adherence and good tolerability, confirming its favorable safety profile during pregnancy.

Overall, these findings underscore the central role of targeted counseling in the prevention of maternal toxoplasmosis. The low uptake of preconception and prenatal screening observed in this cohort represents a missed opportunity for early risk identification and tailored preventive intervention [22,23]. Consistent with previous studies, including those by Martini et al. and Velasco-Velásquez et al., our results support the effectiveness of structured educational interventions in improving preventive behaviors and reducing infection risk [11,21].

Importantly, the dissemination of preventive information within families suggests that individual-level interventions may yield broader public health benefits.

This study has several strengths. First, the case–control design allowed for a direct comparison between pregnant women with *T. gondii* infection and seronegative controls recruited within the same clinical setting, thereby reducing selection bias and enhancing internal validity. Second, the comprehensive assessment of sociodemographic characteristics, dietary habits, environmental and animal-related exposures, and preventive behaviors enabled a multidimensional evaluation of infection risk. Third, data collection was based on standardized questionnaires, ensuring methodological consistency and minimizing inter-observer variability. Finally, the integration of behavioral data with clinical and therapeutic information provides clinically relevant insights for prevention and counseling strategies.

Several limitations should also be acknowledged. The monocentric nature of the study may limit the generalizability of the findings to other geographic or healthcare settings. In addition, behavioral and dietary data were self-reported and therefore subject to recall and social desirability bias, particularly after diagnosis. Moreover, the cross-sectional assessment of exposures precluded causal inference and did not allow for temporal relationships between behaviors and infection to be fully established. The absence of multivariable analyses further limited the ability to fully account for potential confounding factors, such as educational level, rural residence, or antenatal care setting. Consequently, residual confounding remains possible, and some subgroup analyses may have been underpowered.

## 5. Conclusions

This study highlights that *T. gondii* infection during pregnancy is primarily driven by modifiable behavioral and environmental exposures rather than by sociodemographic or obstetric characteristics. Despite comparable baseline awareness of toxoplasmosis, infected women differed significantly from seronegative controls in daily practices related to food consumption, environmental exposure, and animal contact, underscoring a persistent and clinically relevant gap between knowledge and effective prevention.

Importantly, our findings emphasize that counseling should move beyond standardized messages toward a more targeted and individualized approach, tailored to each woman’s lifestyle, environmental context, and specific risk profile. Strengthening preconception and early pregnancy counseling through personalized risk assessment may therefore play a key role in reducing maternal infection and preventing congenital toxoplasmosis.

## Figures and Tables

**Table 1:** Characteristics of the study population.

Characteristic	Cases (*n* 100)	Controls (*n* 101)	*p*-Value
Maternal age (years)	34.36 ± 5.02	33.95 ± 5.63	0.544
Ethnicity			**0.005**
- Caucasian	100	89	
- Asian	0	4	
- African	0	4	
- South Asian	0	4	
- South American	0	0	
Educational level			**0.0085**
- Low-level education	13	5	
- Upper secondary school	42	30	
- High-level education	45	66	
Employment status			0.50
- Employed	70	75	
- Unemployed	30	26	
Residential area			**<0.001**
- Urban	71	92	
- Rural	29	9	
Marital status			1.00
- Cohabiting/Married	89	89	
- Single	11	12	
Parity			0.31
- Nulliparous	72	65	
- Multiparous	28	36	
Method of conception			0.22
- Spontaneous	97	93	
- Assisted reproductive technology (ART)	3	8	
Antenatal care setting			**<0.001**
- Private gynecologist	84	52	
- Hospital-based care	16	49	

**Table 2:** Screening practices and knowledge of toxoplasmosis.

Question	Cases (*n* 100)	Controls (*n* 101)	*p*-Value
Preconception toxoplasmosis screening (IgM/IgG)			0.987
- Performed	31	33	
- Not performed	67	68	
Awareness of toxoplasmosis before pregnancy			0.252
- Yes	81	88	
- No	19	13	
Awareness of toxoplasmosis preventive measures			0.174
- Yes	85	92	
- No	15	9	
Source of information			0.15
- Healthcare professionals	77	71	
- Internet and social media	12	9	
- Family or friends	11	21	
Behavioral changes after awareness of infection			**<0.001**
- Yes	73	92	
- No	27	9	
Recommendation of preventive measures to others			0.14
- Yes	97	91	
- No	3	9	

**Table 3:** Dietary habits.

Question	Cases (*n* 100)	Controls (*n* 101)	*p*-Value
Diet type			0.68 *
- Omnivorous diet	97	99	
- Vegetarian diet	3	2	
- Vegan diet	0	0	
Consumption of raw or undercooked meat			0.97
- Never	82	84	
- Occasionally	15	14	
- Frequently	3	3	
Consumption of cured meats (including ham)			**<0.001**
- Never	25	77	
- Occasionally	43	16	
- Frequently	32	8	
Consumption of unpasteurized milk or dairy products			**0.005**
- Never	69	86	
- Occasionally	25	8	
- Frequently	6	7	
Consumption of fresh vegetables			0.27
- Never	8	13	
- Occasionally	31	37	
- Frequently	61	51	
Consumption of frozen vegetables			0.077
- Never	20	34	
- Occasionally	60	53	
- Frequently	20	14	

* Fisher’s exact test.

**Table 4:** Food purchasing and meal consumption habits.

Question	Cases (*n* 100)	Controls (*n* 101)	*p*-Value
Food purchasing			**0.0023**
- Directly from farmers or livestock breeders	16	5	
- Home-grown products (self-produced)	7	1	
- From supermarkets	77	95	
Frequency of meals consumed outside the home (restaurants, cafés, etc.)			**0.10**
- Never	16	26	
- Occasionally	65	64	
- Frequently	19	11	
Verification of food cooking when eating out			**0.001**
- Never	14	2	
- Occasionally	20	12	
- Frequently	66	87	

**Table 5:** Animal exposure and cat-related behaviors.

Question	Cases (*n* 100)	Controls (*n* 101)	*p*-Value
Contact with farm animals or rural environments			**<0.001**
- Yes	51	24	
- No	49	77	
Cohabitation with cats			**0.0049**
- Yes	34	16	
- No	66	85	
Litter box management			**0.0014**
- Direct handling of litter box	16	8	
- Indirect exposure (handled by others)	45	28	
- No litter box exposure/no cats in the household	39	65	
Feeding cats raw or undercooked meat (occasional or regular)			**0.014** *
- Yes	6	0	
- No	94	101	

* Fisher’s exact test.

## Data Availability

The data presented in this study are available on request from the corresponding author.

## References

[1] Torgerson P.R., Mastroiacovo P. (2013). The global burden of congenital toxoplasmosis: A systematic review. Bull. World Health Organ..

[2] Bénard A., Petersen E., Salamon R., Chêne G., Gilbert R., Salmi L.R., European Toxo Prevention Study Group (EUROTOXO) (2008). Survey of European programmes for the epidemiological surveillance of congenital toxoplasmosis. Euro Surveill..

[3] Dubey J.P., Jones J.L. (2008). *Toxoplasma gondii* infection in humans and animals in the United States. Int. J. Parasitol..

[4] Jacquemard F., Ambroise-Thomas P., Petersen E. (2000). Clinical aspects of infection during pregnancy. Congenital Toxoplasmosis: Scientific Background, Clinical Management and Control.

[5] Robert-Gangneux F., Dardé M.L. (2012). Epidemiology of and diagnostic strategies for toxoplasmosis. Clin. Microbiol. Rev..

[6] Hill D., Dubey J.P. (2002). *Toxoplasma gondii*: Transmission, diagnosis and prevention. Clin. Microbiol. Infect..

[7] Peyron F., L’ollivier C., Mandelbrot L., Wallon M., Piarroux R., Kieffer F., Hadjadj E., Paris L., Garcia-Meric P. (2019). Maternal and Congenital Toxoplasmosis: Diagnosis and Treatment Recommendations of a French Multidisciplinary Working Group. Pathogens.

[8] Tomasoni L.R., Messina G., Genco F., Scudeller L., Prestia M., Spinoni V., Bonfanti C., Prefumo F., Castelli F., Meroni V. (2019). Risk of congenital toxoplasmosis in women with low or indeterminate anti-Toxoplasma IgG avidity index in the first trimester of pregnancy: An observational retrospective study. Clin. Microbiol. Infect..

[9] Wallon M., Peyron F., Cornu C., Vinault S., Abrahamowicz M., Kopp C.B., Binquet C. (2013). Congenital toxoplasma infection: Monthly prenatal screening decreases transmission rate and improves clinical outcome at age 3 years. Clin. Infect. Dis..

[10] Lopez A., Dietz V.J., Wilson M., Navin T.R., Jones J.L. (2000). Preventing congenital toxoplasmosis. MMWR Recomm. Rep..

[11] Martini A., Pietrafesa E., Rondinone B.M., Iavicoli S., D’amelio S., Cavallero S., Bonafede M. (2020). Toxoplasmosis and knowledge: What do the Italian women know about?. Epidemiol. Infect..

[12] Mahfouz M.S., Elmahdy M., Bahri A., Mobarki Y.M., Altalhi A.A., Barkat N.A., Al-Essa H.A., Ageely A.H., Faqeeh N.A., Areeshi N.A. (2019). Knowledge and Attitude Regarding Toxoplasmosis among Jazan University Female Students. Saudi J. Med. Med. Sci..

[13] Qadeer A., An H.Y., Wakid M.H., Hussain S., Asiri M., Alzahrani F.M., Alzahrani K.J., Alsharif K.F., Chen C.C., Ahmad I. (2025). Toxoplasmosis awareness among women: A cross-sectional study on knowledge, attitudes, perceptions, and risk factors. BMC Public Health.

[14] Sviben M., Barbić K., Bogdanić M., Reicher E., Glavaš S., Navolan D., Sanković A., Meštrović T., Mlinarić I., Vlădăreanu S. (2025). Emerging Trends in Toxoplasmosis Seroepidemiology in Childbearing-Aged Women in Croatia, 2015–2024. Pathogens.

[15] Benkacem R., Titaouine M., Mammeri A., Gherissi D.E., Mohamdi N., Chebloune Y. (2025). Cross sectional survey on the prevalence and associated risk factors of toxoplasma infection in pregnant women in Biskra (Southeastern Algeria). Comp. Immunol. Microbiol. Infect. Dis..

[16] Bieńkowski C., Stępień M., Cholewik M., Aniszewska M., Pokorska-Śpiewak M. (2023). Comparison of the prevalence of risk factors for *Toxoplasma gondii* infection among pregnant women in rural and urban areas in Poland. Przegl Epidemiol..

[17] Fernicola F., Colciago E., Capitoli G., Merelli E., Arienti F., Poletti De Chaurand V., Scandella G., Carli A., Ornaghi S. (2025). Limited Knowledge of Toxoplasmosis-Specific Preventive Behaviors in Pregnant Women: A Survey Study in Northern Italy. Int. J. Environ. Res. Public Health.

[18] Smereka J., Szarpak L., Ruetzler K., Schacham Y., Smereka A., Dabrowski M., Terpilowska M., Terpilowski L., Adam I. (2018). A multicenter survey on toxoplasmosis knowledge among pregnant women in Poland (the TOWER study). BMC Pregnancy Childbirth.

[19] Cook A.J., Gilbert R.E., Buffolano W., Zufferey J., Petersen E., Jenum P.A., Foulon W., Semprini A.E., Dunn D.T. (2000). Sources of toxoplasma infection in pregnant women: European multicentre case-control study. European Research Network on Congenital Toxoplasmosis. BMJ.

[20] Kapperud G., Jenum P.A., Stray-Pedersen B., Melby K.K., Eskild A., Eng J. (1996). Risk factors for *Toxoplasma gondii* infection in pregnancy. Results of a prospective case-control study in Norway. Am. J. Epidemiol..

[21] Velasco-Velásquez S., Orozco A.S., Ramirez M., Pachón L., Hurtado-Gomez M.J., Valois G., Celis-Giraldo D., Cordero-López S.S., McLeod R., Gómez-Marín J.E. (2024). Impact of education on knowledge, attitudes, and practices for gestational toxoplasmosis. J. Infect. Public Health.

[22] Mazzilli S., Tourdjman M., Noël H., Maisa A., Villena I., Le-Ray C., ENP2021 study group (2025). Estimating the national seroprevalence of *Toxoplasma gondii* infection in pregnant women, France 2021. Epidemiol. Infect..

[23] Pereboom M.T., Manniën J., Spelten E.R., Schellevis F.G., Hutton E.K. (2013). Observational study to assess pregnant women’s knowledge and behaviour to prevent toxoplasmosis, listeriosis and cytomegalovirus. BMC Pregnancy Childbirth.

